# Datasets for training and validating a deep learning-based system to detect microfossil fish teeth from slide images

**DOI:** 10.1016/j.dib.2023.108940

**Published:** 2023-01-31

**Authors:** Kazuhide Mimura, Kentaro Nakamura

**Affiliations:** a Ocean Resources Research Center for Next Generation, Chiba Institute of Technology, 2-17-1 Tsudanuma, Narashino, Chiba 275-0016, Japan; b Frontier Research Center for Energy and Resources, School of Engineering, The University of Tokyo, 7-3-1 Hongo, Bunkyo-ku, Tokyo 113-8656, Japan; c Department of Systems Innovation, School of Engineering, The University of Tokyo, 7-3-1 Hongo, Bunkyo-ku, Tokyo 113-8656, Japan

**Keywords:** Deep learning, Object detection, Mask R-CNN, Image classification, EfficientNet-V2, Ichthyolith, Machine learning, Artificial intelligence

## Abstract

In this paper, we describe the three datasets that were used to train, validate, and test deep learning models to detect microfossil fish teeth. The first dataset was created for training and validating a Mask R-CNN model to detect fish teeth in the images taken using the microscope. The training set contained 866 images and one annotation file; the validation set contained 92 images and one annotation file. The second dataset was created for training and validating EfficientNet-V2 models; it included 17,400 images of teeth and 15,036 images that contained only noise (particles other than teeth). The third dataset was created to evaluate the performance of a system that combines a Mask R-CNN model and an EfficientNet-V2 model; it contained 5177 images with annotation files for the locations of 431 teeth within the images.

**Specifications Table**

**Table:** 

Subject	Earth and Planetary Sciences
Specific subject area	Computers in Earth Sciences
Type of data	Image annotation file (.json and .xml)
How the data were acquired	All images were generated using a digital microscope, RX-100 (Hirox Co., Ltd.), at the Chiba Institute of Technology. The microscope automatically takes images (referred to as microscope images) at multiple focal depths and generates z-stack images. The image sizes of the first and third datasets were set at 640 × 640 and 1200 × 1200 pixels, respectively. The microscope's magnification was 200x, and each pixel measured 0.96 × 0.96 μm. In the second dataset, regions of particles in the microscope images were cropped without changing the aspect ratio. The images were annotated by one of the authors (KM), a researcher with more than six years of experience working with ichthyoliths.
Data format	Raw
Description of data collection	Dataset 1 (file ``01_dataset_for_ai_ichthyolith'') was created using only the microscope images that included at least one tooth particle. The annotation file indicating the tooth's location in each image is also contained. Dataset 2 (file ``02_dataset_for_efficientnet'') consists of images that are composed of particles trimmed from microscope images. The images are classified into two classes (``Tooth'' and “Noise”). Dataset 3 (file ``03_dataset_for_practical_test'') consists of microscope images and annotations, similar to Dataset 1. However, to measure the performance of the detection system in a more realistic environment, we included all the images taken from a sample in this dataset.
Data source location	1. ODP Site 1179 ­ Institution: International Ocean Discovery Program ­ Latitude and longitude: 41°04.79′ N, 159°57.79′ E 2. IODP Site U1366 ­ Institution: International Ocean Discovery Program ­ Latitude and longitude: 26°03.08′ S, 156°53.67′ W 3. MR15-E01 PC 11 ­ Institution: Japan Agency for Marine-Earth Science and Technology ­ Latitude and longitude: 21°58.27′N, 153°47.75′E
Data accessibility	Repository name: Mendeley Data/Datasets for ichthyolith detection [1] Data identification number: 10.17632/zdpz6m9gzf.1 Direct URL to data: https://data.mendeley.com/datasets/zdpz6m9gzf/1
Related research article	[2] K. Mimura, S. Minabe, K. Nakamura, K. Yasukawa, J. Ohta, Y. Kato, Automated detection of microfossil fish teeth from slide images using combined deep learning models, Applied Computing & Geosciences, 16 (2022). 10.1016/j.acags.2022.100092.

## Value of the Data

• Ichthyoliths, microfossils of fish teeth and denticles, are the only microfossils that commonly occur in pelagic clay. Therefore, they are the key microfossils for constraining the depositional age [3] and evolution in the marine ecosystem [4] of pelagic clay.
• The three datasets are resources for training, validating, and testing a deep learning system to detect microfossil fish teeth (called ichthyoliths). This system was proposed by Mimura et al. [2] and designed to save time and manual work for researchers investigating fossils from deep-sea sediment.
• In this research, the proposed system significantly reduced false positives by combining an object detection model, ``Mask R-CNN'' [5], and an image classification model, ``EfficientNet-V2'' [6]. Consequently, not only researchers who work on microfossils but also researchers who observe minor components in images can potentially use the datasets in evaluating image detection software.
• The datasets can be expanded in the future to create more precise detection systems and increase the number of species that can be identified. Furthermore, when sufficient images of fossil species are accumulated, it will be possible to update the system to automatically identify the species of fossils, which will facilitate further research using ichthyoliths.

## 1 Objective

Biostratigraphy of ichthyoliths was intensively studied in 1970−80s [3] when only grayscale microscope images were available. Recent studies on ichthyoliths [7] also share colored images of ichthyoliths; however, they are taken under reflected light, which is currently used mainly for environmental analysis. Therefore, we created a new dataset of colored images taken under transmitted light for biostratigraphic research.

The datasets add value to the original research paper [2] by helping readers understand through the actual presentation of appropriate images and annotations.

## 2 Data Description

Dataset 1 was used to train and validate the Mask R-CNN [5] model to detect fish teeth. The dataset comprises 866 images for training and 92 for validation, along with the accompanying annotation information. Additionally, it contains only images that include at least one ichthyolith. The contours of fish teeth in the images were annotated using the VGG Image Annotator.

Dataset 2 was used to train and validate the EfficientNet-V2 model to classify the regions detected by Mask R-CNN. The dataset comprises 17,400 images of teeth and 15,036 images of noise (particles other than teeth). Some images were manually cropped from the microscope images, but most were cropped automatically using trained Mask R-CNN models. All the images, whether manually or automatically cropped, were checked and classified manually.

Dataset 3 was used to test the system's total performance proposed in Mimura et al. [2]. This dataset contains microscope images of six glass slides, which were not used in Datasets 1 and 2; it comprises 5177 images and annotation files.

## 3 Experimental Design, Materials and Methods

### 3.1 Dataset 1

Glass slides were prepared from deep-sea sediment samples obtained from the Ocean Drilling Program (ODP) Site 1179, Hole 1179C (41°04.79′ N, 159°57.79′ E, water depth 5564 m). The cores were divided into five lithological units [8], and 11 horizons from the pelagic clay unit (Unit III) were selected as the source for Dataset 1. The glass slides were prepared following the method described by Mimura et al. [2], which is based on the traditional ichthyolith observation method [3], but with some modifications by Sibert et al. [9].

Images of the glass slides were taken by a digital microscope RX-100 (Hirox Co., Ltd.) at the Ocean Resources Research Center for Next Generation, Chiba Institute of Technology. Approximately 1000 square images (-1 mm x 1 mm) were captured from a single glass slide (-24 mm x -36 mm of observation area). From these square images, a human observer selected images that contained at least one ichthyolith. The shutter speed and color balance were fixed throughout the process.

**Fig. 1:**
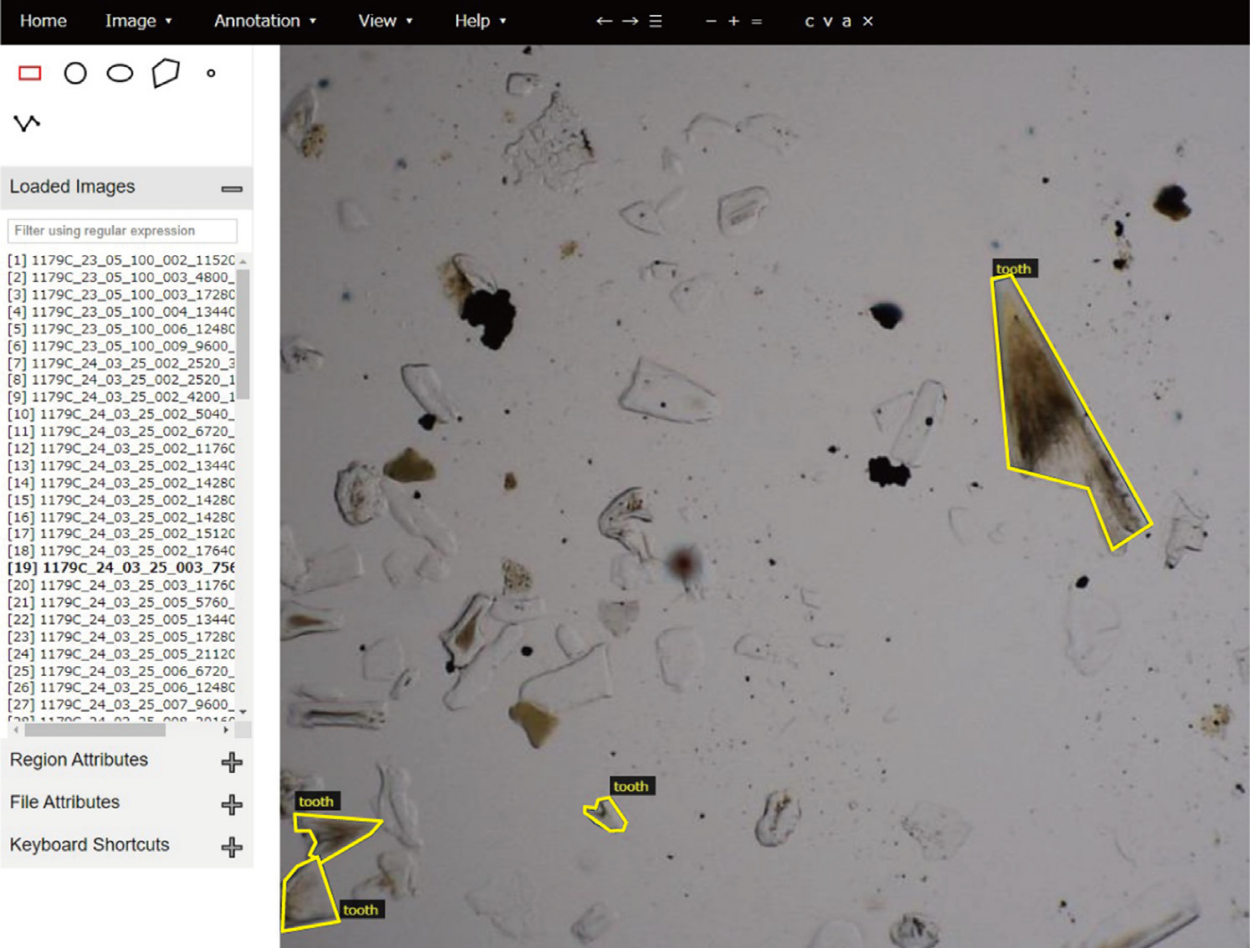
An example annotation from dataset 1.

**Fig. 2:**
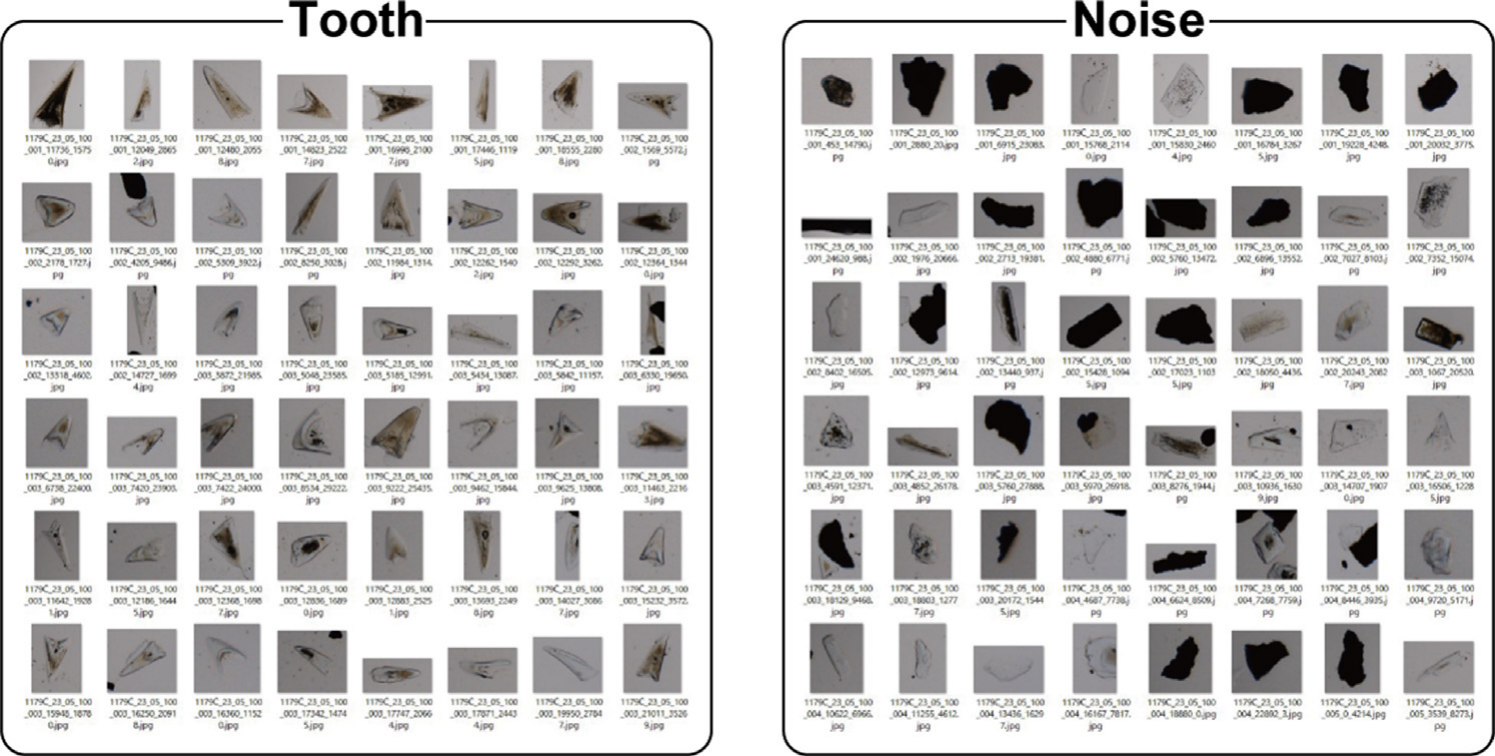
Example images for dataset 2.

For these selected images, the locations of the ichthyoliths were annotated using a VGG Image Annotator [10] as an annotation tool (Fig. 1). The annotation information was formatted as a JSON file containing the image file's name, contour information, and class names of objects in the images.

### 3.2 Dataset 2

Glass slides were prepared using samples from 4 sites—ODP Site 1179, Integrated Ocean Drilling Program (IODP) Sites U1366 and U1370, and MR15-E01 PC11. For Site 1179, the glass slides prepared in Dataset 1 were also used. IODP Site U1366 (Hole U1366C) is located at 26°03.08′ S, 156°53.67′ W, with a water depth of 5130 m. Sediments at Site U1366 were classified into two lithological units [11], and glass slides were prepared from 6 horizons from Unit I (metalliferous pelagic clay). IODP Site U1370 (Hole U1370D) is located at 41°51.12′ S, 153°06.38′ W, with a water depth of 5073 m. Sediments at Site U1370 were classified into three lithological units [11], and the glass slides were prepared from 4 horizons from Unit I (metalliferous clay/metalliferous pelagic clay). MR15-E01 PC 11 is at 21°58.27′N, 153°47.75′E, with a water depth of 5770 m. Sediments at PC11 are all pelagic clay [12]. Glass slides were prepared from two horizons.

Preparing glass slides and taking microscope images followed the same methods for dataset 1. From the microscope images, particles were trimmed and classified into two classes, ``tooth'' and ``noise'' (Fig. 2).

### 3.3 Dataset 3

Glass slides were prepared from ODP Site 1179 (Hole 1179C, Core 24, Section 5, with an interval of 75−77 cm). This sample was not used for datasets 1 and 2. Methods for preparation of glass slides and taking the microscope images followed those for Dataset 1.

This dataset was created to evaluate the performance of the combined detection system; therefore, it utilized all microscope images, including those without ichthyoliths. Locations of ichthyoliths were annotated in the format of Mask R-CNN using VGG Image Annotator.

## Ethics Statements

This work did not involve human subjects, animal experiments, and data collected from social media platforms.

## CRediT authorship contribution statement

**Kazuhide Mimura:** Conceptualization, Methodology, Software, Resources, Data curation, Writing – original draft, Visualization, Funding acquisition, Writing – review & editing. **Kentaro Nakamura:** Conceptualization, Writing – review & editing, Supervision, Funding acquisition, Writing – original draft.

## Declaration of Competing Interest

The authors declare that they have no known competing financial interests or personal relationships that could have appeared to influence the work reported in this paper.

## Data Availability

Datasets for ichthyolith detection (Original data) (Mendeley Data).
